# Efficient derivatization-free monitoring of glycosyltransferase reactions via flow injection analysis-mass spectrometry for rapid sugar analytics

**DOI:** 10.1007/s00216-024-05457-9

**Published:** 2024-08-03

**Authors:** Ulrich Thiele, Chantal Crocoll, André Tschöpe, Carla Drayß, Frank Kirschhöfer, Michael Nusser, Gerald Brenner-Weiß, Matthias Franzreb, Katharina Bleher

**Affiliations:** Institute of Functional Interfaces, Karlsruhe Institute of Technology, Hermann-von-Helmholtz-Platz 1, 76344 Eggenstein-Leopoldshafen, Germany

**Keywords:** Sugar, FIA-MS, Derivatization-free, Enzymatic reaction monitoring

## Abstract

The widespread application of enzymes in industrial chemical synthesis requires efficient process control to maintain high yields and purity. Flow injection analysis-electrospray ionization-mass spectrometry (FIA-ESI–MS) offers a promising solution for real-time monitoring of these enzymatic processes, particularly when handling challenging compounds like sugars and glycans, which are difficult to quickly analyze using liquid chromatography-mass spectrometry due to their physical properties or the requirement for a derivatization step beforehand. This study compares the performance of FIA-MS with traditional hydrophilic interaction liquid chromatography (HILIC)-ultra high-performance liquid chromatography (UHPLC)-mass spectrometry (MS) setups for the monitoring of the enzymatic synthesis of N-acetyllactosamine (LacNAc) using beta-1,4-galactosyltransferase. Our results show that FIA-MS, without prior chromatographic separation or derivatization, can quickly generate accurate mass spectrometric data within minutes, contrasting with the lengthy separations required by LC–MS methods. The rapid data acquisition of FIA-MS enables effective real-time monitoring and adjustment of the enzymatic reactions. Furthermore, by eliminating the derivatization step, this method offers the possibility of being directly coupled to a continuously operated reactor, thus providing a rapid on-line methodology for glycan synthesis as well.

**Graphical Abstract:**

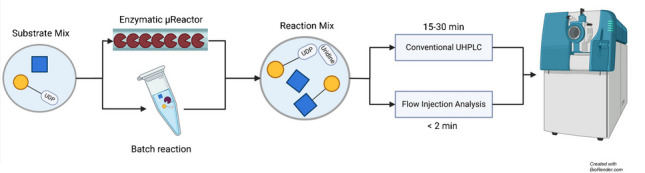

**Supplementary Information:**

The online version contains supplementary material available at 10.1007/s00216-024-05457-9.

## Introduction

Enzymes are now widely used as biocatalysts for the production of basic and fine chemicals [1, 2]. To make production with enzymes sustainable and well-implementable, it is not only important to achieve good yields and purities in the studied reaction but also to employ fast and automated process control. Especially with enzymatic reactions, it is important to quickly determine whether enzymatic activity is decreasing or if there is a change in the reaction mixture composition, to be able to intervene. This is of particular importance for processes that are carried out in continuous flow, as it is increasingly the case [3–5].

Flow injection analysis-electrospray ionization-mass spectrometry (FIA-ESI–MS) has become an established method for high-throughput analysis of samples without prior labeling or derivatization [6–9]. This is achieved by omitting chromatographic separation of analytes before mass spectrometric measurement. Consequently, data from a single sample can be acquired in only a few minutes [10]. This rapid analysis time makes FIA-MS attractive for monitoring enzymatic reactions, as it allows for fast acquisition of information about the system and monitoring of the enzyme activity and reacting promptly to contingencies such as substrate loss or similar issues, thus ensuring optimal reaction conditions and consequently high yields in the biocatalytic transformations [8, 11, 12].

A class of substances that are very difficult to quantify quickly with mass spectrometry due to their physico-chemical properties are sugars and glycans [13]. For example, the quantification of sugars and their respective phosphate derivatives via ESI–MS requires extensive equipment optimization and time-consuming analysis. This is primarily because they are uncharged in solution and have a higher energy requirement for deprotonation in the gas phase [14]. Additionally, measurements must often be conducted in the negative ion mode of mass spectrometry, which typically exhibits lower sensitivity compared to the positive ion mode. This generally reduces the sensitivity of the measurement, although the extent of this reduction greatly depends on the specific analyte [14]. To counteract these effects, a derivatization step is often performed for sugars before MS measurement to increase ionization rates or make detection possible [13, 15]. Another possibility for analysis without prior derivatization is the coupling of the MS method with a suitable liquid chromatography (LC) method, as it has been done in Hong et al. [16]. Here, the use of a hypercarb column, which allows for the sequential detection of sugars over time, was applied. But the analysis time for a single sample, as in many cases using LC–MS methods, often exceeds 30 min due to the extremely shallow solvent gradients needed in order to achieve the separation of different sugars on, as for example, hypercarb or hydrophilic interaction chromatography (HILIC) columns [7, 17–27]. Even with these refined methods, separating isomers like galactose and glucose remains challenging as many methods fail to produce significant changes in their retention times [28–31]. There are specific chromatographic methods that can separate isomers in a very short time; however, these are only suitable for a small number of substrates [32]. Here again, derivatization for example permethylation [33, 34] or a derivatization for an analysis using capillary electrophoresis-mass spectrometry can help to separate complex isomeric structures [35, 36]. Additionally, additives can be added to the solvents to suppress anomer formation, resulting in one signal for a single sugar [30, 37]. Due to all the aforementioned difficulties, a rapid analysis for various sugars is yet not reported, although this would be advantageous, especially for the synthesis of various glycans using enzymes in reactors. Wang et al. were able to achieve a rapid FIA-MS analysis of an aldehyde (5-hydroxymethylfurfural) and suggested in their paper the possibility of transferring this to other aldehydes; however, this has not yet been implemented [38].

The lack of an existing derivatization-free FIA-MS method can certainly also be attributed to the fact that, due to the absence of chromatographic separation, isomers cannot be distinguished, and the lack of derivatization also eliminates any other possibilities of separating the signals. To separate isomers, the combination of ion mobility spectrometry with FIA-MS is therefore often used [39, 40]. However, this configuration is not necessary for the monitoring of reactions catalyzed by enzymes because activated donor sugars and acylated acceptor sugars are needed for those reaction. This results in educts, products, and byproducts with specific masses that can be differentiated purely by their mass to charge ratio.

Another important consideration in enzymatic reaction analyses are matrix effects. Often, biocompatible buffers such as Good’s buffers are needed in enzymatic reactions, which can be separated from the analytes by LC–MS, leading to increased sensitivity of the analytes [41, 42]. However, this is not possible for FIA-MS measurements. Therefore, significant dilutions and sensitive equipment are required to accurately measure in the presence of these matrices or the use of an internal standard is necessary [43, 44].

In this study, we implemented a rapid derivatization-free FIA-MS method for the tracking of enzymatic reactions. In order to validate the method, we compared the accuracy of a HILIC-UHPLC–ESI–MS setup with that of the developed FIA-MS setup. Subsequently, the method was assessed by examining the conversion of N-acetylglucosamine (GlcNAc) with uridine diphosphate galactose (UDP-Gal) to N-acetyllactosamine (LacNAc), catalyzed by beta-1,4-galactosyltransferase. The goal was to develop a method that allows for direct coupling to a continuously operated reactor, thereby enabling on-line analysis of enzymatic glycan production.

## Materials and methods

### Chemicals and buffers

Unless otherwise stated, all chemicals used in this work were purchased from VWR or Sigma-Aldrich/Merck and stored according to the manufacturer’s instructions. Analytical experiments were performed using solvents with LC–MS grade. Buffer components used have cell culture grade and were dissolved in ultrapure water. The concentrations used as well as the pH values of the buffers are given in Table 1. Analytical standards were purchased the highest available purity. For MS experiments, d-glucose-1-^13^C (^13^C-Glc) was used as internal standard. β-1,4-Galactosyltransferase (β1,4GalT1 human recombinant, expressed in HEK 293 cells, 2000 units/mg protein) was purchased from Sigma-Aldrich/Merck and FastAP™ thermosensitive alkaline phosphatase was purchased from Thermo Fisher Scientific. All solutions were prepared with ultrapure water (Milli-Q Gradient, Merck Millipore, Darmstadt, Germany).

**Table 1:** Used buffers with concentrations and adjusted pH values

Buffer solution	Composition	pH
MES	100 mM 2-(N-morpholino)ethanesulfonic acid	5.5
MOPS	100 mM 3-(N-morpholino)propanesulfonic acid	6.5
HEPES	100 mM 4-(2-hydrodyethyl)-1-piperazineethanesulfonic acid	7.5
Tris	100 mM tris(hydroxymethyl)aminomethane	8.5
Glycine	100 mM glycine	9.5

### Mass spectrometry

Mass spectrometry experiments were performed on a TripleTOF 6600 + mass spectrometer (AB SCIEX LLC, Framingham, USA) with a DuoSpray™ ion source using the electrospray ionization mode. Instrument handling and data acquisition was performed using the Analyst Software (version 1.8.1, AB SCIEX LLC, Framingham, USA). For the figures, a smoothing with Gaussian Smoothing 2.0 was used. Data processing, such as extraction of ion chromatograms, calculation of mean spectra, and baseline chromatogram extraction, was performed using the SCIEX OS Software (version 2.1, AB SCIEX LLC, Framingham, USA) and the smoothing settings were set to “high.” A peak width of *m*/*z* ± 0.02 Da was used for all molecules selected in the extracted-ion chromatograms (EIC).

### Operating conditions

Measurements were performed in Product Ion Scan mode in the Analyst Software with negative polarity. In this experiment, a separation of the specific precursor ion in Q1 with targeted fragmentation in q2 followed by the detection of all fragments using the TOF analyzer takes place. For this, the TOF mass range was set between *m*/*z* 60 and *m*/*z* 600 with an accumulation time of 200 ms for each experiment and the mass tolerance was set to 0.1 Da for Q1. The spray voltage was set to − 4500 V. The declustering potential (DP) and collision energy (CE) were optimized for each analyte (Table 2). Ion source gas 1 (nitrogen) was set to 25 psi; gas 2 (nitrogen) was set to 30 psi and curtain gas to 50 psi. The ion source temperature was set to 450 °C. The mass spectrometer was calibrated with APCI Negative calibration solution (AB SCIEX LLC; Framingham, USA) prior to measurements.

**Table 2:** Analyte molecules are listed with the fragment ion used for quantification, as well as the DP (declustering potential) and CE (collision energy) values employed

Analyte	Precursor ion [M-H]^−^ (Da)	Fragment ion (Da)	DP (V)	CE (V)
d-galactose	179.05	119.03	− 20	− 10.0
d-lactose	341.10	179.05	− 25	− 10.0
d-raffinose	503.16	179.05	− 50	− 27.5
d-glucose-1-^13^C	180.06	119.03	− 25	− 10.0
N-acetyl-d-glucosamine	220.08	119.03	− 10	− 15
N-acetyllactosamine	382.14	179.05	− 10	− 15
UDP-a-d-galactose	565.05	323.03	− 80	− 30
uridine	246.06	111.02	− 30	− 15
UDP	402.99	285.27	− 50	− 25

To identify and quantify the analytes, a process method was created with the SCIEX OS-Q software (version 2.1.0) in which a specific fragment ion is selected for the precursor ion, as shown in Table 2.

For each analyte, a calibration curve was created according to the methods used (for the enzymatic reaction only with the FIA-MS method, and for galactose, lactose, and raffinose, both FIA-MS and HILIC-UHPLC-MS were used). Standard solutions of 1 mg/mL in 50:50 (v/v) ACN/H_2_O were diluted to a concentration of 1000 ng/mL in 50:50 (v/v) ACN/H_2_O in two steps in order to prepare a calibration mixture, which was further diluted to the final desired concentrations between 0 and 400 ng/mL and spiked with 100 ng/mL of d-glucose-1-^13^C (in 50:50 (v/v) ACN/H_2_O) as an internal standard (see Table S4 for individual calibration points). For all measurement points, triplicates were taken, except for the 350 ng UDP-Gal measurement point, where a duplicate was used. Blank measurements were conducted but could not be included in the calibration curve, as only background was measured and the software consequently could not divide the area of the analyte by the area of the standard, thus obtaining no value (see Figure S9). The calibration curves used were also not artificially forced through the origin. A detailed listing of the individual parameters of the calibration curves can be found in Table S5.

Calibration curves were obtained with the SCIEX OS Software (version 2.1, AB SCIEX LLC, Framingham, USA) by plotting the ratio of the integral area of the analyte to the integral area of the internal standard over the ratio of the concentration of the analyte to the concentration of the internal standard. Plots for the manuscript were obtained using OriginPro 2023, and analyses of the parameters of the calibration curve were also performed using this software.

### HILIC experiments

For HILIC-UHPLC-ESI–MS experiments, an ExionLC™ system by SCIEX was used. As a chromatographic column, a 1.7-µm HILIC column (particle size 100 Å, 100 × 2.1 mm) from Kinetex was employed. The column oven temperature was maintained at 25 °C. The column was equilibrated for 2 min prior to injection of 10 µL of sample volume. For elution, water and acetonitrile were used for the gradient with a total flow rate of 400 µL/min (Table 3).

**Table 3:** Applied solvent gradient for HILIC-UHPLC-MS measurements

Time (min)	Flow rate (µL/min)	ACN (%)	H_2_O (%)
0.00	400	70.0	30.0
0.50	400	70.0	30.0
3.00	400	60.0	40.0
5.00	400	50.0	50.0
7.00	400	50.0	50.0
8.00	400	70.0	30.0
10.0	400	70.0	30.0

### FIA-MS experiments

For the FIA-MS experiments, a SCIEX M5 Micro LC-TE with an autosampler (PAL 3 CTC) was used. It offers two binary gradient pumping systems (G1 or G2), G1 for low flow rates between 1 and 10 µL/min and G2 for high flow rates between 20 and 200 µL/min. FIA–MS measurements were performed with a flow rate of 50 µL/min. For this, G2 was connected to the injection valve instead of G1 to secure a constant isocratic flow at this rate (see Scheme S1). The other connections on the injection valve remain the same as described by SCIEX for FIA-MS measurements. A 5-µL loop was used in full-loop injection mode. Before injection, the needle was dipped twice in organic and aqueous solvent and washed thrice. After injection, the syringe was washed twice with organic and aqueous solvents, respectively. The M5 was connected to the mass spectrometer 6600 + using a capillary from Phenomenex with dimensions of 50 µm × 1000 mm and SecurityLINK fittings.

### Sample preparation for galactose, lactose, and raffinose and quantification

The buffer solutions were individually prepared in ultrapure water and utilized for dissolving galactose, lactose, and raffinose to a concentration of 10 mM. This concentration was subsequently diluted to 1 mM using the corresponding buffer solutions. Following this, the samples were further diluted in a 50:50 mixture of ACN/H_2_O at ratios of 1:10,000; 1:20,000; and 1:50,000, resulting in concentrations of 18.1 ng/mL, 12.1 ng/mL, and 3.6 ng/mL for galactose, lactose, and raffinose samples, respectively. The internal standard (d-glucose-1-^13^C at 100 ng/mL) was introduced during the final dilution step as previously outlined.

### Enzymatic reactions

The enzymatic reaction was performed in 100 µL of 100 mM HEPES buffer at pH 7.5 with 25 mM KCl and 6.2 mM MnCl_2_. The substrates N-acetylglucosamine and UDP-galactose were added from stock solutions in a concentration of 5 mM and 6.2 mM, respectively. Commercial alkaline phosphatase was added in a concentration of 5 U. The commercial β-1,4-galactosyltransferase (2000 U/mg) was dissolved in 50 µL ultrapure water, equaling 2 U/µL. The reaction was started by adding 10 µL of the β-1,4-galactosyltransferase solution to the reaction mixture.

The reaction was incubated at 30 °C and 1000 rpm. Samples of 5 µL each were taken after 2, 5, 10, 15, and 30 min and transferred to 995 µL of 50:50 ACN/H_2_O. The reaction was stopped by heating the sample at 70 °C for 5 min.

Samples were further diluted to a final concentration of 1:50,000, spiked with 100 ng of d-glucose-1-^13^C and analyzed via FIA-MS as previously described.

### Statistical analyses and simulation of time course experiment

To compare the collected values between the HILIC-UHPLC-MS method and the data obtained from the FIA-MS method, various statistically relevant values were calculated. The calculations were performed using Excel. To estimate the similarity of the results with HILIC- and FIA-MS, we performed a two-sided paired *t*-test. We obtained *p*-values for the individual dilutions of the substances larger than 0.4. This *p*-value corresponds to the probability of obtaining the two samples if both were drawn from the same probability distribution. The comparatively high *p*-value is congruent with the null hypothesis that HILIC- and FIA-MS measurements are equivalent. (The corresponding *p*-value was obtained by referring the *t*-value to the *t*-distribution with the degrees of freedom, in this case 14.) Averages and standard deviations were calculated with the corresponding formulas. The coefficient of variation and the accuracy were calculated using the below stated formulas:

$$\text{Coefficient of variation }(\text{\%})= \frac{\text{average}}{\text{standard deviation}}\times 100$$

$$\text{Accuracy }\left(\text{\%}\right)= \frac{\text{average }-\text{ true value}}{\text{true value}}\times 100$$

The usefulness of the data of the developed FIA-MS method for kinetic parameter extraction is demonstrated for the described enzymatic reaction of β-1,4-galactosyltransferase. For this, the time course of the decreasing concentration of the substrate UDP-galactose (UDP-Gal) is simulated assuming the validity of (i) a pseudo single-substrate mechanism, or (ii) a bi-substrate mechanism forming a temporary ternary-complex with the enzyme in order to react to the products. In the case of mechanism (i), the reaction rate is given by the simple Michaelis–Menten equation.

$$v= -\frac{d\left[S\right]}{dt}= \frac{{k}_{\text{cat}}\bullet \left[E\right]\bullet \left[S\right]}{{K}_{\text{M}}+ \left[S\right]}$$

In the case of mechanism (ii), the reaction rate can be described by:

$$v= -\frac{d\left[S\right]}{dt}= {k}_{\text{cat}}\bullet \left[E\right]\bullet \frac{\left[{S}_{1}\right]}{{K}_{\text{M},1}+ \left[{S}_{1}\right]} \bullet \frac{\left[{S}_{2}\right]}{{K}_{\text{M},2}+ \left[{S}_{2}\right]}$$

However, because in our case only one time course with almost equivalent concentrations of the two substrates is used for parameter extraction, the fitting algorithm cannot distinguish between ${K}_{\text{M},1}$ and ${K}_{\text{M},2}$. In consequence, only an effective value $K{'}_{\mathrm M}$ can be determined, assuming $K{'}_{\mathrm M}=K_{\mathrm M,1}=K_{\mathrm M,2}$. The parameter extraction was carried out minimizing the root mean square error (RMSE) between the simulated and the experimental time course of the substrate GlcNAC applying the Solver Add-in of Excel.

## Results and discussions

Our goal was to establish a rapid method for assessing the enzymatic activity of glycosyltransferases. Assays for these types of reactions are scarce and, if they exist, often rely on UDP cleavage, which is a very nonspecific reaction. A conventional setup with MS would involve the use of a HILIC-UHPLC-MS method coupled to the MS, which still involves analysis times of 10 min. Therefore, we opted for a FIA-MS setup where the analysis time is 1–2 min.

**Fig. 1:**
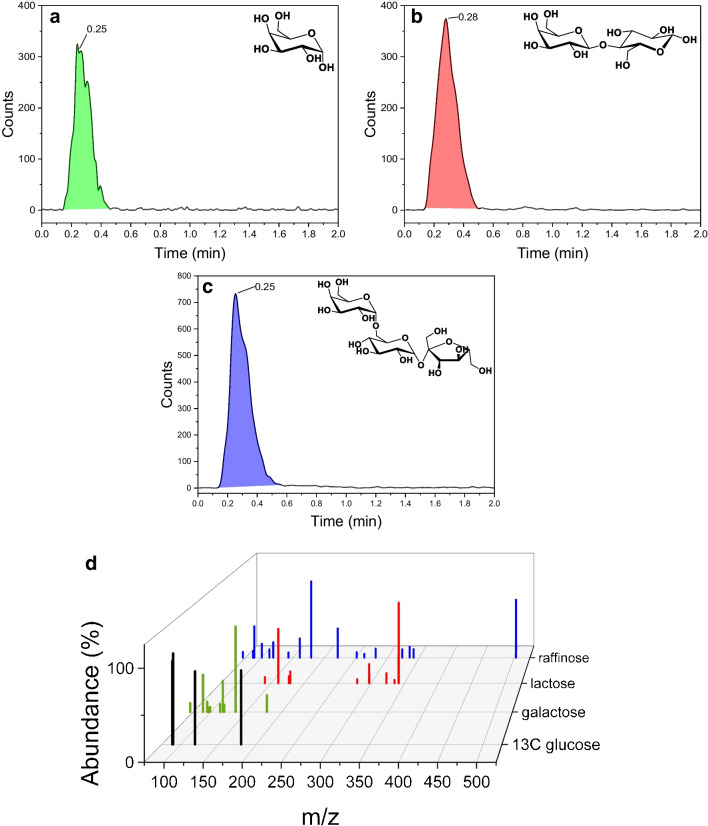
Elution profile of **a** galactose, **b** lactose, and **c** raffinose. **d** PIS of all mentioned analytes and d-glucose-1-.^13^C (*m*/*z* range of 60–600, 85 cycles, 200 ms accumulation time, the abundance of the fragment peaks was normalized in respect to the highest abundance of the respective PIS)

The substrates galactose, lactose, and raffinose were measured to evaluate the setup by measuring sugars of various sizes. In Fig. 1, the elution peaks of each substance are plotted. The spray parameters were optimized for the applied flow of 50 µL/min of 50:50 ACN/H_2_O (Table 2) for each sugar. With these settings, a sharp elution peak was achieved for all three substances, with over 95% of the signal intensity observed within less than 0.5 min (see Fig. 1a–c). The quantification of the analytes was carried out using a product ion scan (PIS), where the fragment ions were selected in such a way that they stood out clearly from the background signals. The transitions used for each analyte are summarized in Table 1, along with the optimal spray conditions. The overlaid PIS spectra of the individual components are depicted in Fig. 1d, including the internal standard (d-glucose-1-^13^C), which was added to compensate for matrix effects that eventually occur later. With those settings, a calibration curve was recorded for each analyte (see SI).

### Comparison of HILIC-UHPLC-MS and FIA-MS

To assess the reliability of the FIA-MS results, an initial comparison was made using a UHPLC-ESI–MS system with a HILIC column. For this comparison, we selected different buffer systems as well as three saccharides with varying sizes to determine the effect of different matrices and molecular weights and how well d-glucose-1-^13^C could be used as an internal standard to compensate for said effects. This is particularly relevant in the context of enzymatic reactors where product streams can have high buffer and salt concentrations as well as significant solution variability. Consequently, calibrations were performed using d-glucose-1-^13^C as an internal standard to compensate for variable ionization rates, without prior incorporation of the buffer solutions in the calibration process. Furthermore, multiple dilutions were analyzed to determine their effect on matrix effects and hence on the analyte signal and reproducibility.

**Fig. 2:**
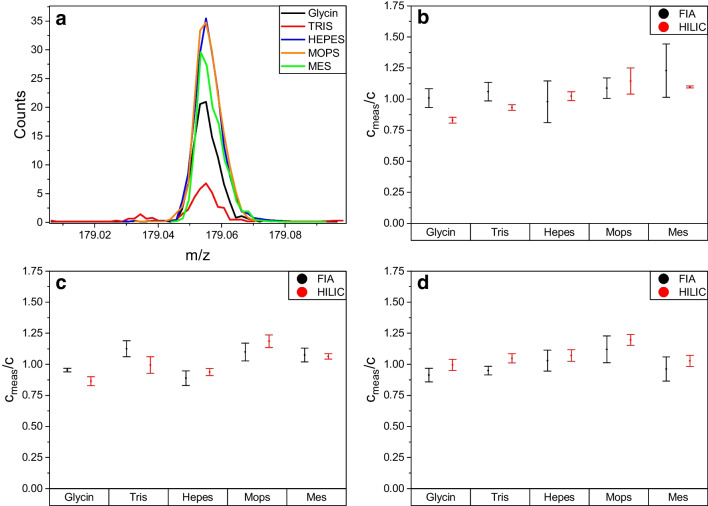
Comparison of **a** the EICs of a 50 ng/mL raffinose solution in different buffers (100 mM of the respective buffer). 1 mM raffinose in 100 mM buffer and diluted **b** 1:10,000, **c** 1:20,000, and **d** 1:50,000. Shown are the results conducted with FIA-MS and HILIC-UHPLC-MS. Results are normalized on target concentrations

Following the initial experiments, identical tests were subsequently performed using the described FIA-MS setup. Both methods used individual calibrations with the same calibration mixture in 50:50 (v/v) ACN/H_2_O without any added buffers. In Fig. 2, different dilutions of a 1 mM raffinose solution in various buffers (100 mM) were measured (see SI Figure S3 and Figure S4 for galactose and lactose). This 1 mM solution was either diluted: 1:10,000; 1:20,000; or 1:50,000 prior to measurement, resulting in target concentrations of 18.1, 12.1, and 3.6 ng/mL, respectively. For better comparability, all measurement results were normalized to the theoretically expected concentration.

As can be seen in Fig. 2a with the extracted ion count of a 50 ng/mL raffinose solution, the obtained ion yield is strongly dependent on the buffer used. Surprisingly, the only phosphate-containing buffer, MOPS, along with HEPES, achieved the highest signal. The fluctuations in ionization are compensated for by the internal standard d-glucose-1-^13^C, which is subject to the same matrix effects. In Fig. 2b–d, the comparison of the HILIC-UHPLC-MS and FIA-MS measurements with different dilutions is shown for raffinose (see SI Figure S3 and Figure S4 for galactose and lactose and detailed statistical analysis in Tables S1–S3).We observed that across all substrates and concentrations both methods, HILIC- and FIA-MS, yield very similar results (*p* > 0.4, Student’s *t*-test, see “Materials and methods” and SI). Hence, both methods are suitable for conducting the analysis. The results from both setups reveal two opposing dynamics. At high sample concentrations, the significant matrix effects of the buffer are manifested as large fluctuations at individual measurement points, seen in bigger coefficients in variation (CV) for raffinose (CV FIA/HILIC for dilution 1:10,000: 13.2%/11.8%; 1:20,000: 9.9%/11.7%; 1:50,000: 9.9%/7.4%). On the other hand, at high dilutions and low concentrations, detection becomes difficult due to the detection limit of the method, in this case resulting in an overestimation of the actual concentration for both the HILIC- and the FIA-MS method with a deviation of about 25%. This is particularly noticeable for the mono-and disaccharide, galactose and lactose, where the CV values are higher. Additionally, measurement accuracy improves with increasing substrate mass (average accuracy of the measurements across all dilutions and both methods used: raffinose: 3.5%; lactose: 10.9%; galactose: 15.1%, see the “Materials and methods” section and Tables S1–S3) since the final concentration is in a more favorable range of the calibration curve. Overall, the use of an internal standard in both the HILIC-UHPLC-MS and FIA-MS methods facilitates the generation of consistent and reproducible results, effectively mitigating the influences of diverse matrices.

Based on these findings, it can be concluded that the FIA-MS method is suitable for fast analysis of reaction mixtures from enzymatic processes without significant loss of information in comparison to the HILIC-UHPLC-MS, provided that an internal standard is used.

### Reaction control of an enzymatic reaction

**Fig. 3:**
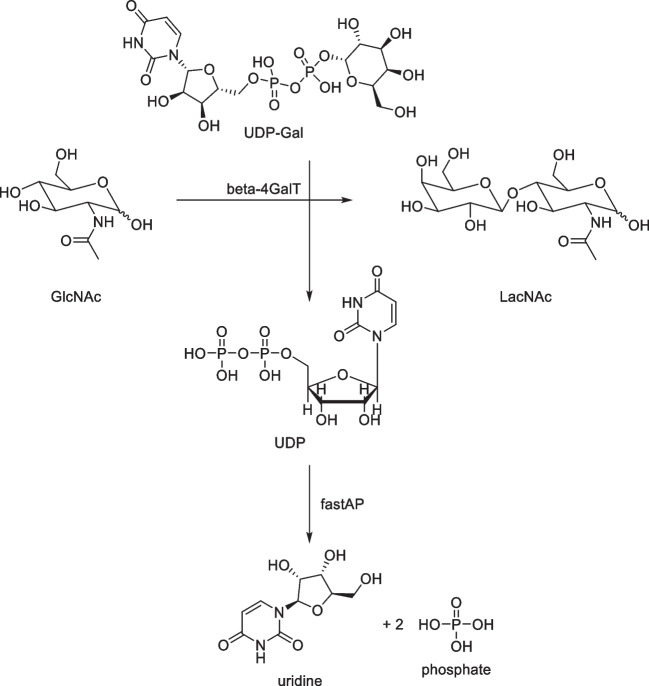
Illustration of the studied reaction: beta-1,4-galactosyltransferase (β1,4GalT1) converts N-acetylglucosamine (GlcNAc) and UDP-galactose (UDP-Gal) to N-acetyllactosamine (LacNAc). The byproduct UDP formed in the process is broken down to uridine and free phosphates by the alkaline phosphatase (FastAP), as it inhibits the transferase

Following the verification of adequate accuracy of the FIA-MS setup, an enzymatic reaction involving the beta-1,4-galactosyltransferase was investigated. This enzyme catalyzes the transfer of galactose from uridine-diphosphate-galactose (UDP-Gal) to N-acetylglucosamine (GlcNAc), creating N-acetyllactosamine (LacNAc) (Fig. 3), which is a component of many glycoproteins and is also found in the structure of human milk oligosaccharides (HMOs). As a second enzyme, alkaline phosphatase (FastAP) was added to break down the byproduct UDP into uridine and free phosphates, as UDP inhibits galactosyltransferase activity [45]. The reaction was carried out in 100 mM HEPES buffer with 25 mM KCl and 6.2 mM MnCl_2_ at pH 7.5 as described above.

**Fig. 4:**
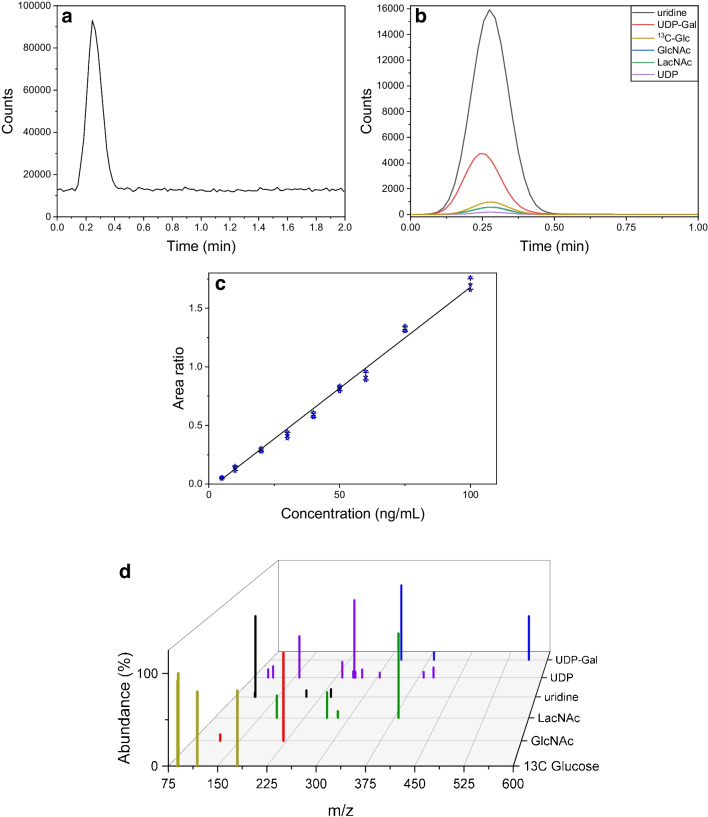
Mass signals and elution profiles obtained in FIA-MS measurements of the enzymatic reaction: **a** elution profile of the reaction mixture with a concentration of 50 ng/mL (each substance, calibration mixture); **b** EIC of all analytes; **c** exemplary calibration curve for GlcNAc (*r*^2^ = 0.994, MES: 5.317 (ng/mL).^2^). **d** Mass spectrum in product ion scan mode (*m*/*z* range of 60–600, 85 cycles, 200 ms accumulation time, the abundance of the fragment peaks was normalized in respect to the highest abundance of the respective PIS)

Before the reaction could be investigated, the MS calibration for the reaction mixture was performed as described in the “Materials and methods” section. All reactants, products, and byproducts were measured from 2.5 to 400 ng/mL and the corresponding calibration curves for all analytes were created (see Fig. 4d and Figure S8, Tables S4 and S5). This concentration range was chosen, because the samples from the enzyme reaction were diluted 1:50,000 in 50:50 (v/v) ACN/H_2_O and spiked with 100 µL (equals 100 ng) of d-glucose-1-^13^C in 50:50 (v/v) ACN/H_2_O prior to measurement. The dilution of the reaction mixture results in calculated concentrations of 22.12 ng/mL GlcNAc and 70.22 ng/mL UDP-Gal. The high dilution rate was chosen as it yielded the most stable values in the experiments described previously, yet it causes a consistent slight overestimation of the concentrations (see Fig. 2). The total ion chromatogram (TIC) of a calibration mix with 50 ng/mL for all components is depicted in Fig. 4a. The EICs for all calibrated analytes are shown in Fig. 4b. Consistent with previous findings, sharp elution peaks were observed, with no analyte signals detected beyond 0.5 min post-injection. An exemplary calibration curve for GlcNAc is given in Fig. 4c, with additional calibration curves available in the Supplementary Information. All obtained calibration curves have a coefficient of determination, *R*-squared, greater than 0.99. In addition, the mean squared error (MSE) was determined, which is the largest for the calibration curve of GlcNAc shown here, at 5.317 (ng/mL)^2^. In 7 out of 11 cases, however, the MSE is significantly below 1, which, along with a good coefficient of determination, generally indicates good accuracy. Figure 4d displays an overlay of all product ion scans, indicating that complete fragmentation was achieved as evidenced by the disappearance of all precursor signals.

**Fig. 5:**
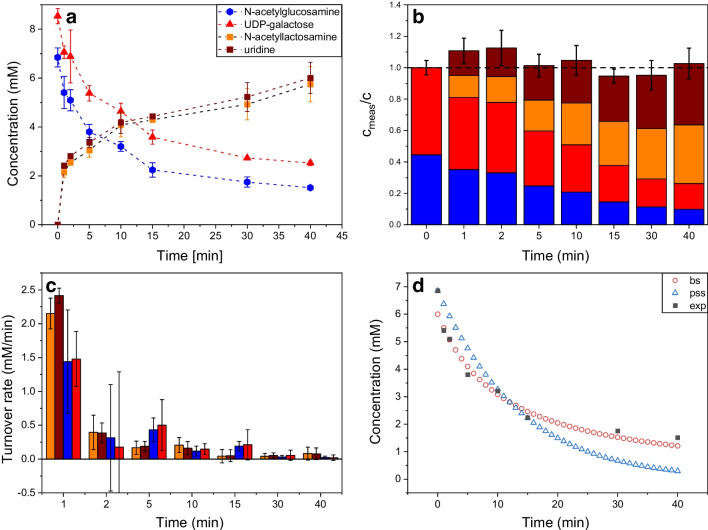
**a** Temporal progression of the enzymatic reaction of beta-1,4-galactosyltransferase measured by FIA-MS. **b** Time course of the normalized mass balance calculated by reference to the measured total educt concentration at time *t* = 0. 20 U of beta-1,4-galactosyltransferase and 5 U of FastAP™ were incubated at 30 °C and 1000 rpm in 100 mM HEPES buffer with 25 mM KCl and 6.2 mM MnCl_2_ at pH 7.5 for 40 min. FastAP™ was added to degrade UDP into uridine and free phosphates. Reaction was stopped by heat shock at 70 °C. **c** Turnover rates in mM per minute for reactants and products. **d** Simulation of the time course experiment of UDP-Gal (squares, exp) assuming a pseudo single substrate mechanism (triangles, pss) and a bi-substrate mechanism (circles, bs)

The enzymatic reaction was carried out as described above in a 100-µL reaction volume at 30 °C and 1000 rpm. At certain time intervals, a 5-µL sample was taken, stopped by thermal deactivation, diluted to 1:50,000, and analyzed using FIA-MS. The dynamic progression of the reaction is illustrated in Fig. 5a, which shows the parallel degradation of the substrates and the concurrent accumulation of the product LacNAc, as well as the byproduct uridine. Notably, the intermediate UDP was not detected, aligning with expectations given the inclusion of FastAP in the reaction mixture to break down the inhibitory UDP [45] into uridine and phosphates. The near parallel increase of LacNAc and uridine indicates that the concentration of FastAP was sufficient to instantly degrade UDP as it was created in conjunction with LacNAc, as expected for a 1:1 stoichiometric conversion.

Ideally, the molar amount of the consumed substrates (GlcNAc + UDP-Gal) equals the molar amount of the synthesized product (LacNAc) and byproduct (uridine). During the 40-min reaction time, 5.11 mM ± 0.06 mM GlcNAc and 6.02 mM ± 0.10 mM UDP-Gal (start concentrations of 5 mM and 6.2 mM, respectively) were converted into 5.75 ± 0.71 mM LacNAc and 6.00 ± 0.63 mM uridine. The mass balance for each time point can also be seen in Fig. 5b, and the corresponding turnover rate for each analyte is displayed in Fig. 5c. The results shown in Fig. 5b are normalized in respect to the measured initial concentrations of the educts, to compensate for the mentioned consistent overestimation of the values. This allows to assess the constancy of the mass balance over time, based on the initial quantities of reactants used. The inaccuracy caused by the high dilution was deliberately accepted in order to reduce matrix effects and thus achieve constant ion yields in contrast. The goal with this setup was not to achieve a particularly sensitive or precise method, but to obtain reliable results in the shortest possible time in order to develop a process analytical tool that allows almost real-time control of enzyme reactions in future. Figure 5b shows that the values of the normalized mass balance match the ideal value of 1 within their error margin at almost all of the measured time points. An even better proof of the validity of the measured data results from the fact that the changes in the individual concentrations of the reactants closely follow the stoichiometry of the enzymatic reaction. The generated amounts of product and byproduct correspond to the expected 1:1 ratio. At the same time, the bars indicating the normalized educt concentrations drop by the same amount from step to step. In consequence, the fast measurement of all involved reactants using the developed FIA-MS method allows a much more detailed control of the progress of the reaction than would be possible with the conventional approach of using photometric assays to follow only a single educt or product. For example, the occurrence of an unwanted side reaction could be detected directly from an increasing violation of the mass balance. In addition, the individual concentration data would allow conclusions to be drawn as to whether the side reaction occurs with one of the educts or the product formed.

To demonstrate how the measurement of detailed concentration time courses of reactants of enzymatic reactions could also be used to extract kinetic parameters, we conducted simulations of the time course of the UDP-Gal concentration assuming different reaction mechanisms. Note, since the experiment was carried out at only one initial concentration of reactants, the simulations are not able to deliver a rigorous analysis of all kinetic parameters of this multi-substrate reaction. Rather, the simulations conducted as described in the “Materials and methods” section serve to illustrate the basic procedure of parameter extraction. Assuming a pseudo single substrate mechanism resulted in a clear deviation between the observed and the simulated concentration course, even after fitting the kinetic parameters by RMSE minimizing. The probable reason for this is given by the fact that we have a stoichiometric reaction between two reactants, neither of which is in excess, which is why the assumption of pseudo single-substrate is not valid. As can be seen in Fig. 5d, assuming a bi-substrate mechanism with both substrates limiting the reaction rate results in a much better agreement between simulated and experimental data. Because the time course indicates that even the initial substrate concentrations are in a range resulting in substrate limitations, ${k}_{\text{cat}}$ and ${K}_{\text{M}}{\prime}$ cannot be extracted independently, but only the so-called catalytic efficiency ${k}_{\text{cat}}/{K}_{M}{\prime}$ resulting in a value of 6400 s^−1^ M^−1^, which is in the typical range for moderately active enzymes [46].

The ability to perform the analysis without prior removal of the buffer or the need for derivatization significantly shortens the analysis time. From sample collection, thermal deactivation, through a dilution step to measurement, it takes about 10 min until the results are obtained, making it an extremely fast at-line analytics tool. In terms of future studies, this method is also suitable for the on-line analysis of a continuously operated reactor, as it makes the purification and derivatization steps redundant. Here, the flow of the reactor mixture only needs to be diluted and spiked with an internal standard to be able to monitor the reactor activities on-line.

## Conclusion

We have shown that the use of a non-time-resolved FIA-MS method for the quantitative analysis of sugars without derivatization does not result in a loss of information compared to a conventionally used HILIC-UHPLC-ESI–MS method, when an internal standard is used to cancel out matrix effects. Subsequently, this method was applied for activity monitoring of a sugar transferase. With thermal deactivation, a dilution step and subsequent measurement, results can be obtained within 10 min since no derivatization or sample purification step was necessary, making it a fast, derivatization-free at-line analytic. Due to its robustness, we envision that with appropriate instrument setups, on-line monitoring of enzymatic reactions in continuous operation is also possible. However, it is generally advisable to use an internal standard even for a continuous enzymatic reaction, as fluctuations in the composition of the medium, such as different salt, buffer, or substrate concentrations, can occur. These effects can also be taken into account without the need to recalibrate the devices.

## Supplementary Information

Below is the link to the electronic supplementary material.

Supplementary file1 (PDF 634 KB)
