# Supplementary material for: Efficient derivatization-free monitoring of glycosyltransferase reactions via flow injection analysis-mass spectrometry for rapid sugar analytics
Source: Anal Bioanal Chem. 2024 Aug 3;416(23):5191–203. doi: 10.1007/s00216-024-05457-9 (PMC11377506; doi:10.1007/s00216-024-05457-9)
Supplement: Supplementary file 1 — Supplementary file1 (PDF 634 KB) [file 216_2024_5457_MOESM1_ESM.pdf]

Efficient derivatization-free monitoring of glycosyltransferase reactions via flow injection analysis- mass spectrometry for rapid sugar analytics

Ulrich Thiele[a], Chantal Crocoll[a], André Tschöpe[a], Carla Drayß[a], Frank Kirschhöfer[a], Michael Nusser[a], Gerald Brenner-Weiß, [a] Matthias Franzreb[a] and Katharina Bleher[a]

a) Institute of Functional Interfaces, Karlsruhe Institute of Technology, Hermann-von-Helmholtz-Platz 1, 76344, Eggenstein-Leopoldshafen, Germany

Supporting Information

Table of Contents

FIA-MS experiments 3

Product Ion Scans for FIA -MS 4

Ion counts for HILIC-UHPLC-ESI-MS 5

Comparison FIA-MS/HILIC-UHPLC-ESI-MS 6

Ion counts for FIA-MS 9

Calibration Curves 10

FIA-MS experiments

For the FIA-MS experiments, a SCIEX M5 Micro LC-TE with Autosampler (PAL 3 CTC) was used. It offers two binary gradient pumping systems (G1 or G2), G1 for low flow rates between 1 µL/min to 10 µL/min and G2 for high-flow rates between 20 µL/min to 200 µL/min. FIA-ESI-MS measurements were performed with 50 µL/min flow rates. For this, G2 was connected to the injection valve instead of G1 to secure a constant isocratic flow at this rate (see Scheme 1). The other connections on the injection valve remain the same as described by SCIEX for FIA-MS measurements. It should be noted that when using only G2, the temperature sensor of the M5 must be moved from G1 to G2. A 5 µL loop was used in full-loop injection mode. Before injection the needle was dipped twice in organic and aqueous solvent and washed thrice. After injection the syringe was washed twice with organic and aqueous solvent respectively.

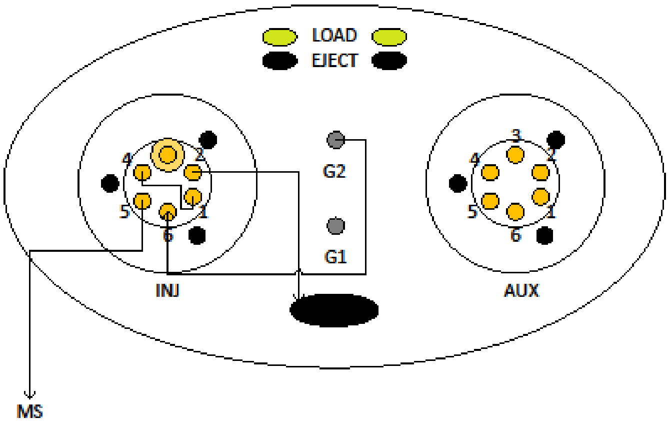

Scheme S1: Illustration of the configuration of the valves for an FIA-MS experiment on the M5. Since higher flow rates of 50 µL/min were used in the experiment, G2 was connected to port 6 of the injection valve, not G1.

Product Ion Scans for FIA -MS

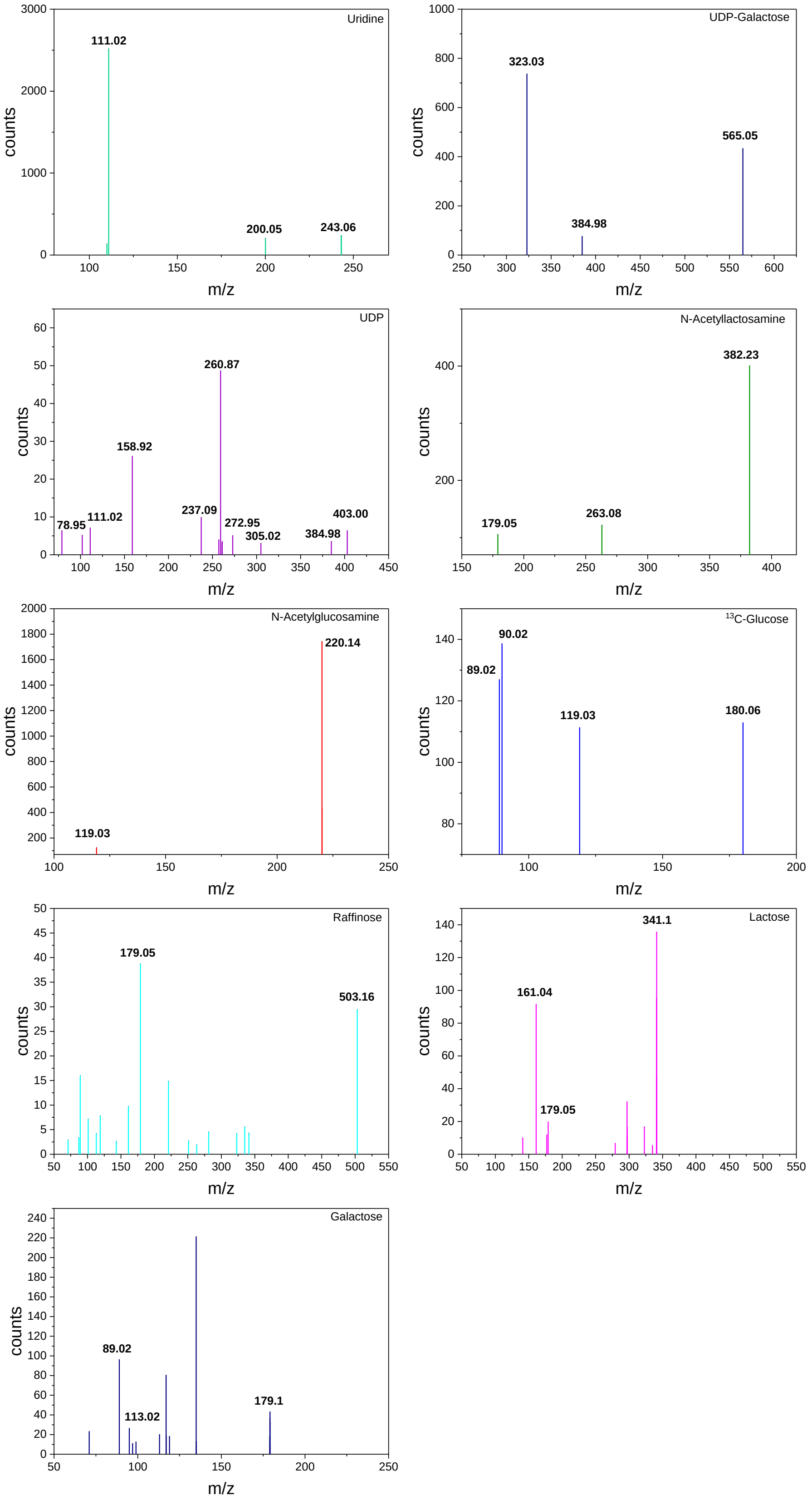

Figure S1: Product Ions Scans for all in this paper analyzed compounds (all compounds have a concentration of 50 ng/mL, expect ^{13}C-D-Glucose with a concentration of 100 ng/mL). The name of the respective analyte is specified in the top right corner of the respective PIS.

Ion counts for HILIC-UHPLC-ESI-MS

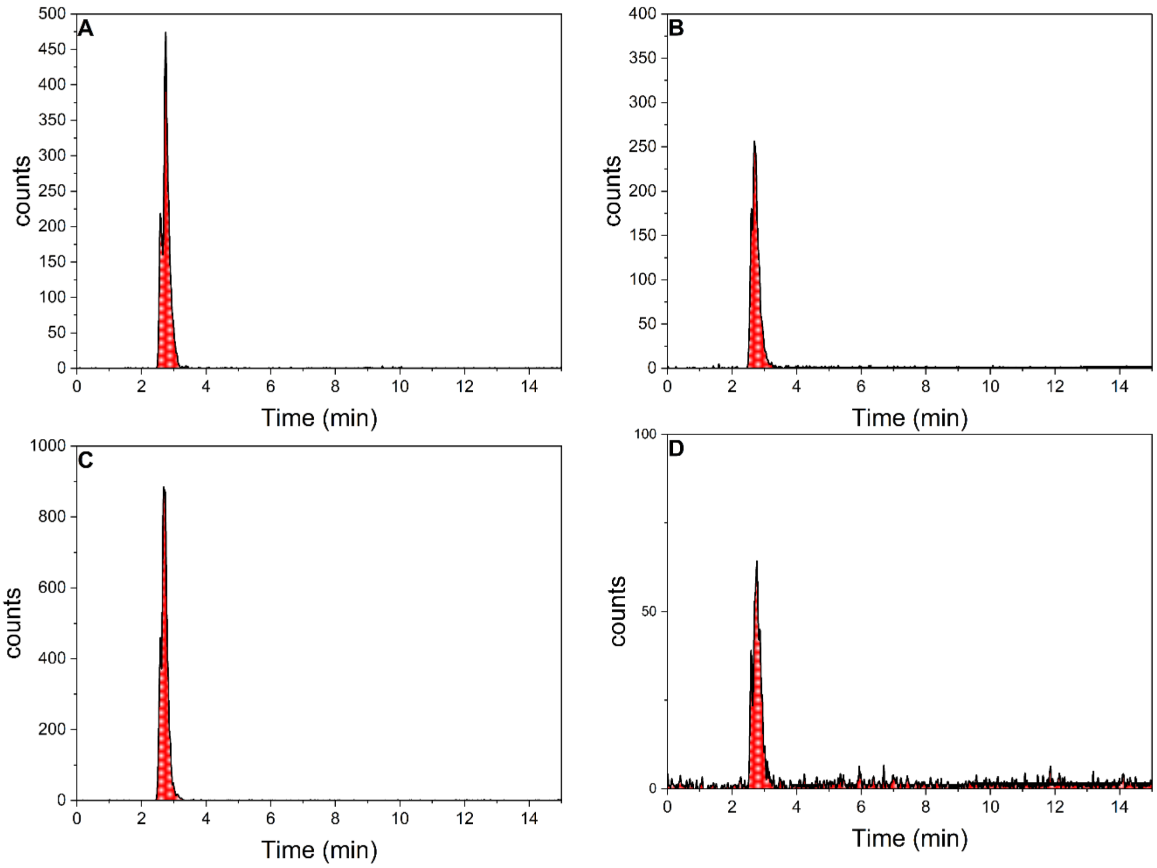

Figure S2: EICs for A: 1-^{13}C glucose, B: lactose, C: raffinose, D: galactose measured with the HILIC-setup. Sample concentrations were at 50 ng/mL (note: Although the LC program used lasted 10 minutes, MS data was collected for 15 minutes. This is an artifact of the software used to control the 6600 MS and the micro-LC M5.).

Comparison FIA-MS/HILIC-UHPLC-ESI-MS

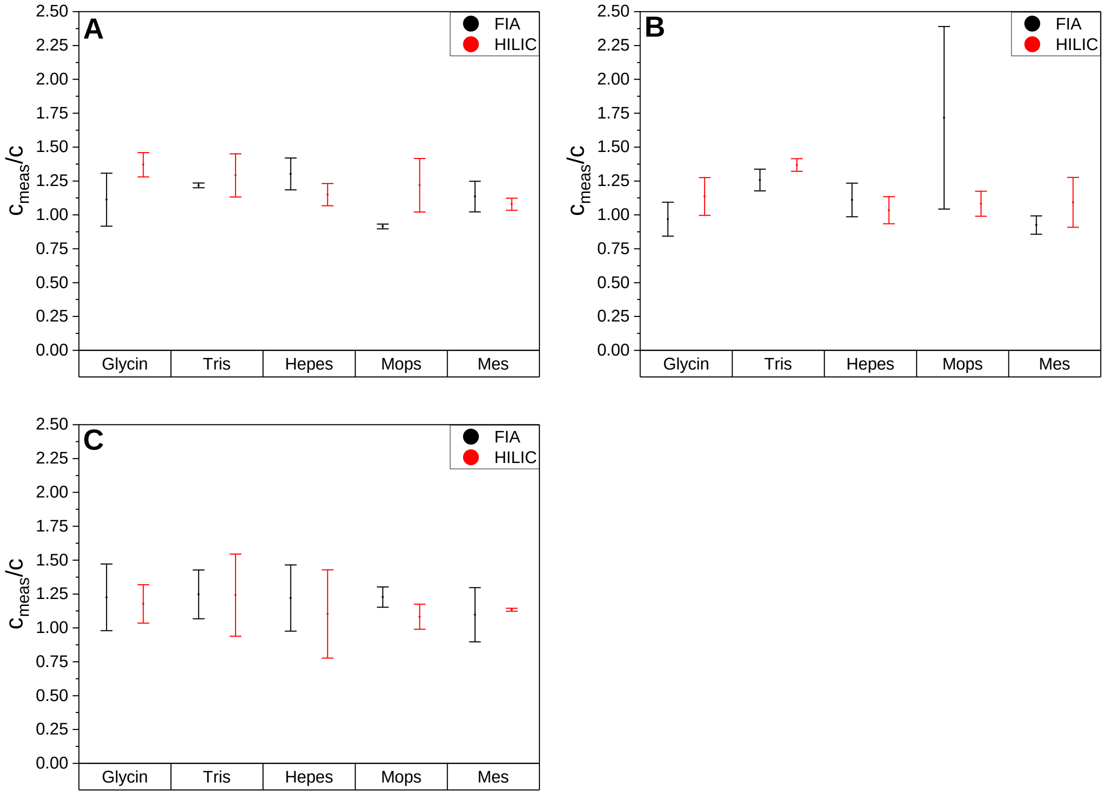

Figure S3: Comparison of the HILIC-UHPLC-MS and FIA-ESI-MS methods for Galactose using various buffers and different dilutions. Results are normalized on target concentrations.

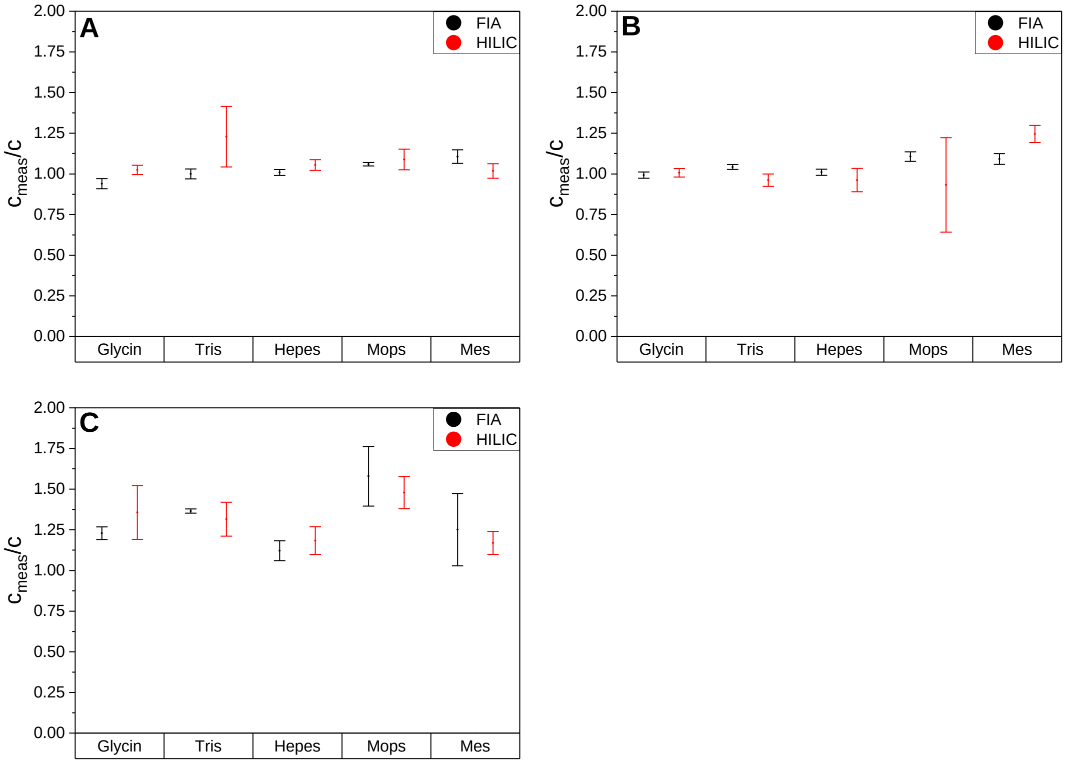

Figure S4: Comparison of the HILIC-UHPLC-MS and FIA-ESI-MS methods for Lactose using various buffers and different dilutions. Results are normalized on target concentrations.

Statistical Anayses

As described in the Methods section, a statistical analysis of the data between FIA-MS and HILIC-UPLC-MS was performed. The calculated values are presented in Table 1-3. The formulas used are listed in the Methods section.

Table S1 Measurements of raffinose using the two methods, FIA-MS and HILIC-UHPLC-MS, in various buffers and dilutions. The measurements were normalized to the expected concentration. The values from the statistical analysis are also displayed.

**raffinose**						
**dilution**		10000		20000		50000
**method**	FIA	HILIC	FIA	HILIC	FIA	HILIC
**Glycin**	1.01	0.81	0.94	0.89	0.90	1.02
	0.93	0.83	0.96	0.88	0.86	0.94
	1.08	0.85	0.97	0.82	0.97	1.02
**TRIS**	1.04	0.91	1.07	1.07	0.99	1.01
	1.15	0.95	1.11	0.93	0.93	1.08
	1.00	0.94	1.20	0.98	0.93	1.06
**HEPES**	0.89	1.05	0.95	0.97	1.04	1.07
	1.17	0.99	0.83	0.92	1.11	1.12
	0.88	1.03	0.88	0.93	0.94	1.02
**MOPS**	1.13	1.19	1.15	1.17	1.16	1.16
	1.00	1.02	1.02	1.24	1.21	1.19
	1.14	1.22	1.13	1.14	1.00	1.24
**MES**	1.47	1.09	1.03	1.04	1.03	1.06
	1.15	1.10	1.06	1.09	1.00	1.04
	1.07	1.10	1.14	1.06	0.85	0.98
**T-TEST**		0.119656		0.500688		0.001314
**p-value**		0.906456		0.62437		0.99897
**average**	1.07	1.02	1.03	0.98	0.99	1.06
**standard deviation**	0.141183	0.121208	0.102387	0.115632	0.098485	0.077505
**Coefficient of Variation (%)**	13.24292	11.83207	9.911679	11.74908	9.949446	7.304066
**accuracy (%)**	6.61	2.44	3.299	-1.582	-1.015	6.112

Table S2 Measurements of lactose using the two methods, FIA-MS and HILIC-UHPLC-MS, in various buffers and dilutions. The measurements were normalized to the expected concentration. The values from the statistical analysis are also displayed.

**lactose**						
**dilution**		10000		20000		50000
**method**	FIA	HILIC	FIA	HILIC	FIA	HILIC
**Glycin**	0.93	1.03	0.98	1.04	1.26	1.55
	0.91	1.05	0.99	0.99	1.19	1.27
	0.97	0.99	1.01	0.99	1.24	1.26
**TRIS**	0.96	1.08	1.05	0.95	1.38	1.22

	1.01	1.17	1.05	0.93	1.36	1.43
	1.02	1.44	1.03	1.00	1.36	1.30
**HEPES**	1.00	1.09	1.02	0.90	1.18	1.14
	1.03	1.02	0.99	1.04	1.06	1.13
	0.99	1.05	1.02	0.94	1.12	1.28
**MOPS**	1.05	1.10	1.10	1.16	1.49	1.37
	1.07	1.14	1.14	1.03	1.46	1.51
	1.06	1.02	1.08	0.61	1.79	1.56
**MES**	1.15	0.97	1.12	1.19	1.47	1.13
	1.11	1.05	1.10	1.25	1.25	1.13
	1.06	1.04	1.06	1.30	1.03	1.25
**T-TEST**		0.09778		0.52114		0.84967
**p-value**		0.923493		0.658115		0.409804
**average**	1.02	1.05	1.05	1.00	1.26	1.27
**standard deviation**	0.061258	0.107732	0.048465	0.160202	0.189995	0.145769
**Coefficient of Variation (%)**	5.995157	10.25059	4.622804	15.96417	15.06842	11.5169
**accuracy (%)**	2.179	5.098	4.838	0.351	26.088	26.57

Table S3 Measurements of galactose using the two methods, FIA-MS and HILIC-UHPLC-MS, in various buffers and dilutions. The measurements were normalized to the expected concentration. The values from the statistical analysis are also displayed.

**galactose**						
**dilution**	10000		20000		50000	
**method**	FIA	HILIC	FIA	HILIC	FIA	HILIC
**Glycin**	1.31	1.44	0.89	0.98	1.50	1.06
	1.10	1.27	1.11	1.24	1.03	1.14
	0.92	1.40	0.90	1.19	1.15	1.34
**TRIS**	1.20	1.31	1.20	1.34	1.43	0.97
	1.23	1.44	1.23	1.42	1.24	1.19
	1.23	1.12	1.35	1.34	1.07	1.57
**HEPES**	1.17	1.22	1.25	1.03	1.04	0.89
	1.37	1.17	1.02	0.93	1.12	1.48
	1.37	1.06	1.06	1.13	1.50	0.94
**MOPS**	0.93	1.44	1.48	1.06	1.31	1.06
	0.92	1.06	2.48	1.18	1.16	1.18
	0.90	1.15	1.19	1.00	1.21	1.00
**MES**	1.16	1.05	0.87	1.30	1.23	1.13
	1.01	1.13	1.00	0.98	1.19	1.15
	1.23	1.05	0.90	1.00	0.87	1.13
**T-TEST**		0.182513		0.618943		0.489986
**p-value**		0.857797		0.508614		0.631733
**average**	1.17	1.17	1.11	1.13	1.19	1.13
**standard deviation**	0.159583	0.146232	0.384122	0.153935	0.171356	0.183121

**Coefficient of Variation (%)**	13.68228	12.49741	34.52564	13.56399	14.39568	16.19995
**accuracy (%)**	16.635	17.01	11.257	13.488	19.033	13.038

Ion counts for FIA-MS

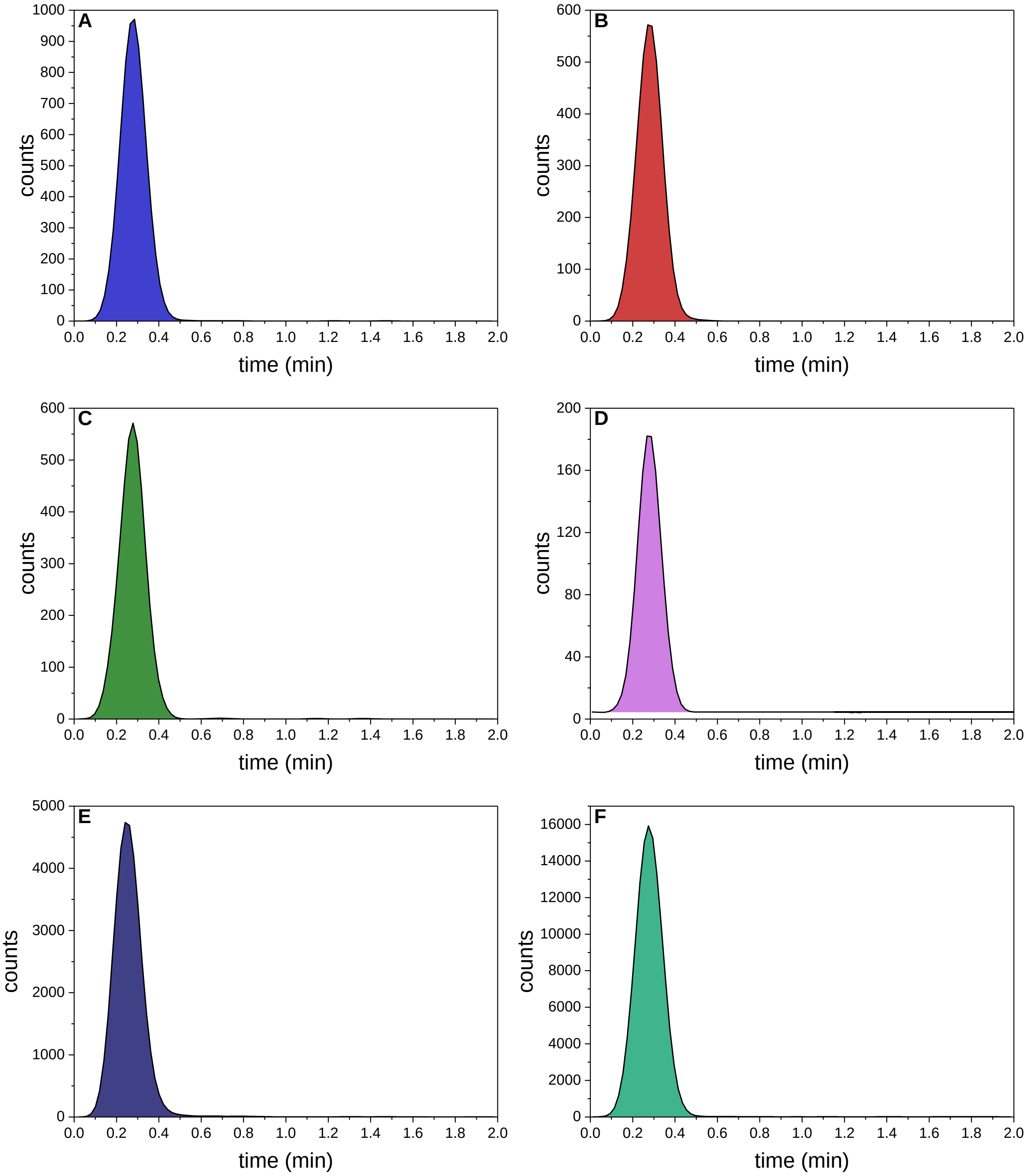

Figure S5: EICs for FIA -MS of A: 1-^{13}C-D-Glucose (100 ng/mL), B: GlcNAc, C: LacNac, D: UDP, E: UDP-Gal and F: uridine conducted with FIA-ESI-MS at a concentration of 50ng/mL.

Calibration Curves

Calibrations between 0 – 50 ng/mL were prepared for FIA-MS and HILIC Experiments for galactose, lactose and raffinose.

For the kinetic measurement of the beta-1,4-galactosyltransferase, a calibration curve between 0 – 400 ng/mL was prepared. Ion signal intensities of fragments of uridine-diphosphate were not reproducible between 0-100 ng/mL. Accordingly, it is reasonable to assume that the limit of detection was reached in this area.

All measurements were carried out trice except for UDP-Gal 350 ng, here the experiment was only carried out twice. Blank measurements were conducted but could not be included in the calibration curve, as only background was measured and the software consequently could not divide the area of the analyte by the area of the standard, thus obtaining no value (see Figure S9)

Table S4: Stock solutions were prepared by dissolving the respective analytes in 50:50 ACN/H_2O to a concentration of 1 mg/mL. These were further diluted to 1000 ng/mL in the case of the analytes and 10000 ng/mL in the case of the internal standard. To each calibration mix, 10 µL of internal Standard was added, resulting in 100 ng/mL ^{13}C-D-Glucose.

Final Concentration of Analytes [ng/mL]	Added solvent (50:50 ACN/H_2O) [µL]	Added Stock solution of Analytes (1000 ng Stock) [µL]	Added Volume of ISTD 1-^{13}C-D-Glucose [µL]	Total Volume [µL]
**0**	990	0	10	1000
**2.5**	987.5	2.5	10	1000
**5**	985	5	10	1000
**10**	980	10	10	1000
**20**	970	20	10	1000
**25**	965	25	10	1000
**30**	960	30	10	1000
**40**	950	40	10	1000
**50**	940	50	10	1000
**60**	930	60	10	1000
**70**	920	70	10	1000
**80**	910	80	10	1000
**90**	900	90	10	1000
**100**	890	100	10	1000
**125**	865	125	10	1000
**150**	840	150	10	1000
**175**	815	175	10	1000
**200**	790	200	10	1000
**225**	765	225	10	1000
**250**	740	250	10	1000
**300**	690	300	10	1000
**350**	640	350	10	1000
**400**	590	400	10	1000

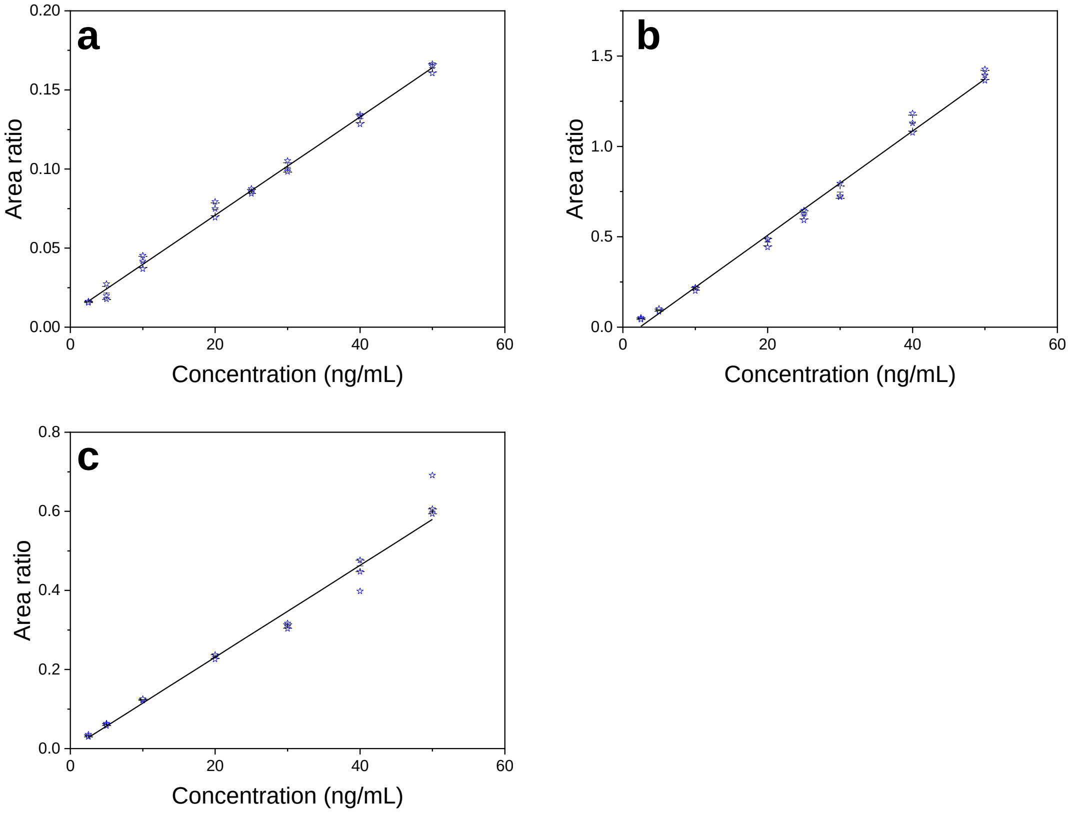

Figure S6: Calibration curves for HILIC-U(H)PLC-ESI-MS A: galactose(r^2=0.9951); B: lactose (r^2=0.9908) and C: raffinose (r^2=0.9915).

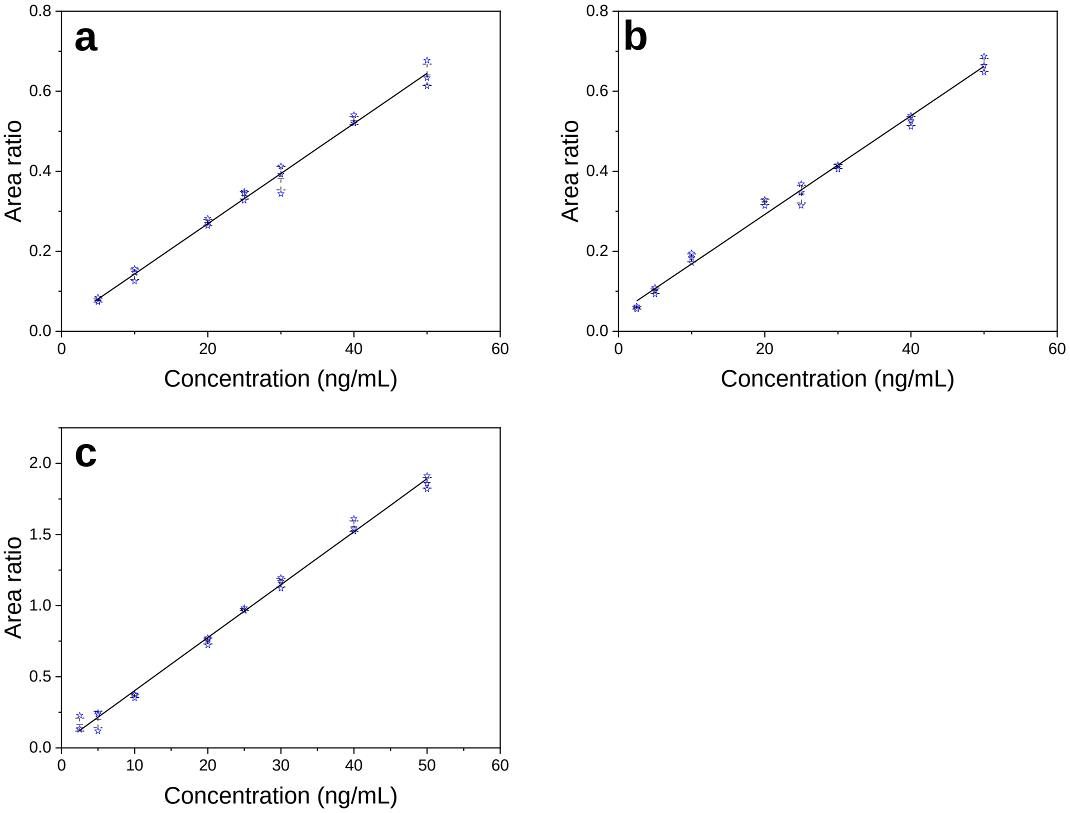

Figure S7: Calibration curves for FIA-ESI-MS A: galactose(r^2=0.9905); B: lactose (r^2=0.9903) and C: raffinose (r^2=0.9941).

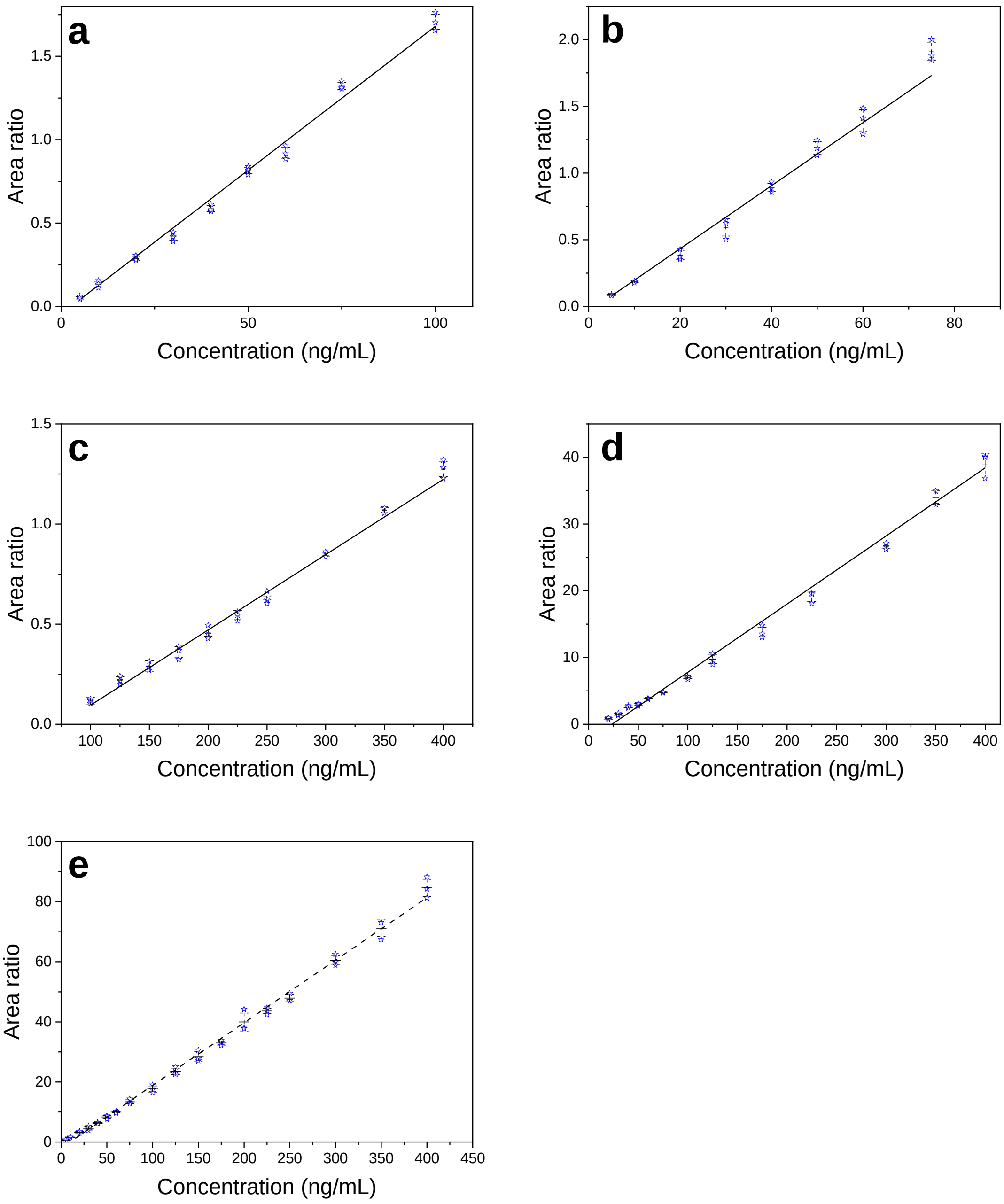

Figure 8: Calibration curves for the enzymatic reaction for the FIA -MS setup. A: GlcNAc (r^2=0.9940); B: LacNAc; (r^2=0.9911); C: UDP (r^2=0.9944); D: UDP-Gal (r^2=0.9908) and E: uridine (r^2=0.9974).

The key parameters of the calibration curves are listed in the table below.

Table S5: Summary of the key statistical parameters of the calibration curves.

	slope	intercept	r^2	Residual sum of squares	MES $(ng/mL)^2$	Method
**galactose**	0.01254 ± 2.81293E-4	0.01804 ± 0.00834	0.99053	0.00686	0.0004	FIA

	0.00311 ± 4.65583E-5	0.00864 ± 0.00129	0.99508	2.85127E-4	0.00001	HILIC
**lactose**	0.01234 ± 2.61007E-4	0.04513 ± 0.00724	0.99026	0.00896	0.0004	FIA
	0.01149 ± 2.69296E-4	2.52262E-4 ± 0.00691	0.99075	0.00591	0.00035	HILIC
**raffinose**	0.03724 ± 6.10515E-4	0.02992 ± 0.01694	0.99412	0.04903	0.002	FIA
	0.0288 ± 5.69981E-4	-0.06726 ± 0.01581	0.99146	0.04273	0.0019	HILIC
**GlcNAc**	0.01724 ± 5.04651E-4	-0.0448 ± 0.0154	0.99404	21.43171	5.317	FIA
**LacNAc**	0.0236 ± 9.15058E-4	-0.03887 ± 0.00932	0.99106	83.59826	3.572	FIA
**UDP-Gal**	0.10211 ± 0.00297	-2.41019 ± 0.57888	0.99078	19.5459	1.777	FIA
**UDP**	0.00376 ± 9.94531E-5	-0.28138 ± 0.02445	0.99444	0.00687	0.001	FIA
**uridine**	0.20856 ± 0.00265	-2.02183 ± 0.49079	0.99743	28.41659	1.777	FIA

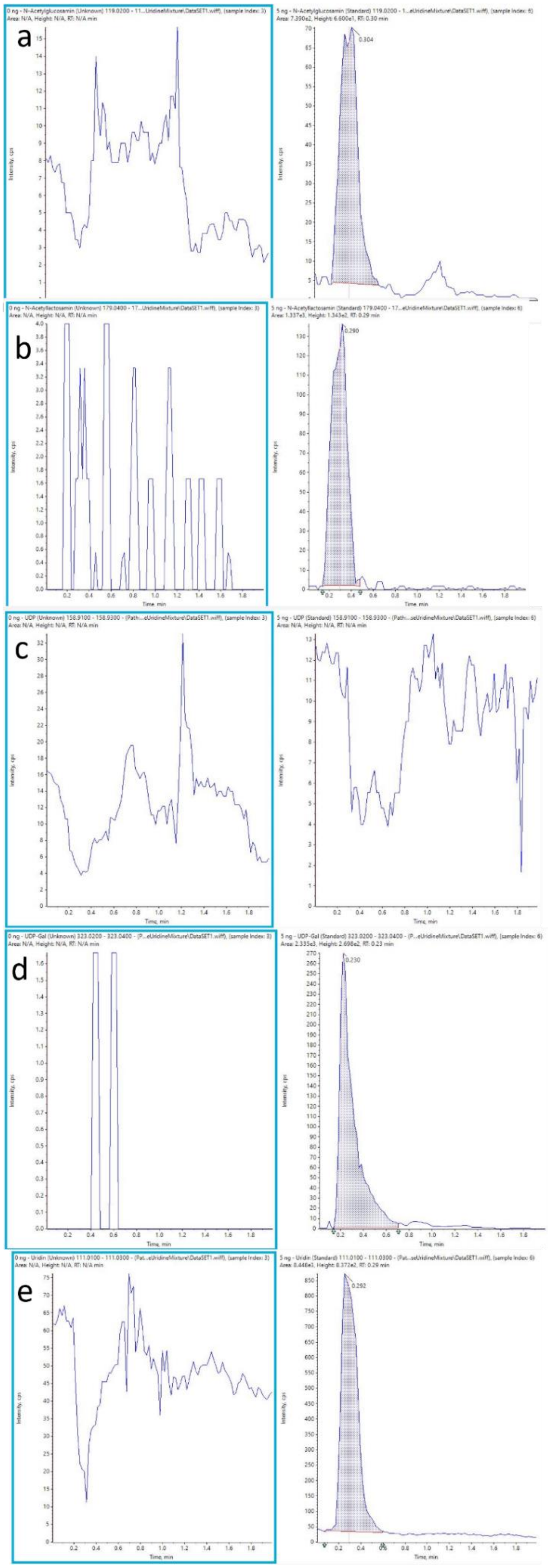

Figure S9: EICs of the respective analytes at 0 and 5 ng a: GlcNAc; b: LacNAc; c: UDP; d: UDP-Gal, e: uridine.
